# Increased serum CXCL16 is highly correlated with blood lipids, urine protein and immune reaction in children with active nephrotic syndrome

**DOI:** 10.1186/1746-1596-9-23

**Published:** 2014-01-24

**Authors:** Junhui Zhen, Qian Li, Yanji Zhu, Xiujun Yao, Li Wang, Aihua Zhou, Shuzhen Sun

**Affiliations:** 1 School of Medicine, Shandong University, Jinan, China; 2 Department of Pediatric Nephrology and Rheumatism and Immunology, Provincial Hospital Affiliated to Shandong University, Jinan, China; 3 Department of Pathology, Shandong University School of Medicine, Jinan, China; 4 Department of Pediatric Nephrology, Provincial Hospital Affiliated to Shandong University, Jingwu Road 324, Jinan 250021, China

**Keywords:** Nephrotic syndrome, CXCL16, Proteinuria, Cellular immunity

## Abstract

**Background:**

Primary nephrotic syndrome (NS) is a common disease in children. Lipid nephrotoxicity and cellular immune dysfunction are known features of this disease. Recently, CXCL16 was found to participate in inflammation and mediate cellular uptake of lipids. Here, we investigated the involvement of CXCL16 in the occurrence and development of primary NS.

**Methods:**

Serum CXCL16, blood lipids and albumin, 24-hour urine protein, interferon-γ and immune cells were detected in 25 children with steroid sensitive NS during their active nephrotic and remissive stages. Twenty healthy children served as the control group.

**Results:**

Levels of serum CXCL16, blood lipids, interferon-γ and CXCR6+ T cells were significantly increased and albumin and NK cell number were significantly decreased in the active status group compared with remissive status and control groups. Correlation analysis showed that serum CXCL16 was positively correlated with blood lipids, 24-hour urine protein, interferon-γ and CXCR6+ T cells but negatively correlated with albumin in patients with active NS.

**Conclusion:**

Serum CXCL16 was increased in patients with active NS and correlated with blood lipids, urine protein and immune and inflammation responses, suggesting that CXCL16 may serve as a useful index or biomarker for disease activity in children with nephrotic syndrome.

**Virtual slides:**

The virtual slide(s) for this article can be found here: http://www.diagnosticpathology.diagnomx.eu/vs/1120468411154766.

## Introduction

Nephrotic syndrome (NS) is a nonspecific disorder of the kidney stemming either directly from kidney disease (primary NS) or indirectly via conditions in other parts of the body affecting the kidney (secondary NS) [1,2]. NS is characterized primarily by high proteinuria (>3 g/dL), hypoalbuminemia and edema resulting from damage to the glomerular filtration membrane, causing the kidney to leak large amounts of plasma protein from the blood into the urine. This trait is typically accompanied by dyslipidemia with elevated plasma cholesterol and triglycerides. In vitro studies have demonstrated that the major components of lipids, such as low-density lipoprotein (LDL), oxidized LDL (oxLDL) and very low-density lipoprotein, could directly stimulate mesangial cells to proliferate and secrete inflammatory factors, including IL-6, TGF-β, MCF-1, connective tissue growth factor and PDGF. In addition, LDL and oxLDL could promote the activation of renal immune cells, subsequently upregulating NF-κB activity and hastening the release of inflammatory factors [3]. Therefore, kidney inflammation may be the result of a hyperlipidemia-induced influx of inflammatory mediators. However, the mechanism of lipid deposition in the kidney, the initial step for the development of primary NS, is largely unknown.

Chemokines are a class of small secreted proteins involved in inflammation and the immune response. The chemokine superfamily consists of nearly 50 chemokines and 20 chemokine receptors [4], and the interaction of chemokines and their receptors is a key mediator of inflammation and arteriosclerosis. C-X-C motif chemokine ligand 16 (CXCL16), first described by Matloubian [5] and Wilbanks [6], exists in transmembrane-bound and soluble forms. Transmembrane-bound CXCL16 acts as both a cell surface adhesion molecule and a novel scavenger receptor. Furthermore, transmembrane-bound CXCL16 may be released to its soluble form upon digestion by a disintegrin and metalloproteinase (ADAM) protein, specifically ADAM10 and ADAM17. Soluble CXCL16 can recruit activated immune cells that express CXCR6, the receptor of CXCL16 [7-9], and mediate immune response-related inflammation [10-12]. In recent years, CXCL16 was found to participate in the development of atherosclerosis. The major pathological feature of atherosclerosis is the formation of foam cells, derived from either macrophages or smooth muscle cells, and CXCL16 is expressed on the surface of macrophages, arterial smooth muscle cells and vascular endothelial cells. Transmembrane-bound CXCL16 can also combine with oxLDL and mediate the cellular uptake of lipids. Thus CXCL16 may be involved in the formation of foam cells. Taken together, the results of these studies imply that CXCL16 may, through the mediation of both lipid deposition and immune and inflammatory responses, be involved in the development of primary NS. However, the degree to which CXCL16 participates in the occurrence of primary NS in children is largely unknown.

This study aims to reveal the function of CXCL16 in the occurrence of childhood primary NS by monitoring levels of CXCL16 protein in the serum of children with primary NS and assessing any correlation with interferon-γ (IFN-γ), 24-hour urine protein, serum albumin and lipid metabolism. Through the data, we hope to establish a new theoretical basis by which to improve understanding and treatment of this disease.

## Materials and methods

### Subjects

Of 50 children with primary nephrotic syndrome admitted in our department from January 2010 to June 2011, 25 with complete data and off steroid treatment were retrospectively enrolled in this study. This group consisted of 16 males and nine females ranging in age from 1.5 to 13 years (median = 5.0 years).

Patients were tested during both active and remissive stages over the course of at least three months. Twenty age-matched healthy children from a local school or kindergarten were chosen as the healthy controls. This group consisted of 12 males and eight females ranging in age from 3 to 12 years (median = 6.5 years). We confirmed that these children were without current or past kidney disease, infectious disease or kidney-related family history. At the time of testing, no patient or control was taking any immunomodulating drug (e.g., cyclosporine A, cyclophosphamide, levamisole or MMF) which could affect the immunologic parameters studied.

There was no significant difference in age or gender between the NS groups and control group (P > 0.05). This study was approved by the ethics committee of Provincial Hospital Affiliated to Shandong University, and written informed consent for participation was obtained from the parents of those who participated.

### Definitions

Primary NS was defined by heavy proteinuria (urinary protein > 50 mg/kg/day), hypoalbuminemia [serum albumin < 2.5 g/dL] and hypercholesterolemia (serum cholesterol > 5.72 mmol/L), with or without edema. Initial treatment was comprised of 2 mg/kg/day (maximum 60 mg/day) of prednisone given orally in three divided doses for four weeks followed by therapy on alternate days for another four weeks. The daily dose was then tapered down for 4–7 months and finally stopped.

The active stage of NS was defined as increased urinary protein excretion (Albustix > 2+ for at least three consecutive days or > 50 mg/kg/day) and serum albumin concentration of < 25 g/L. Remission was defined as serum albumin concentration of > 35 g/L and normal protein excretion (Albustix trace or negative for at least three consecutive days). “Steroid-sensitive” was defined as disappearance of proteinuria (negative for three consecutive days) following four weeks of prednisone treatment. Patients who still had proteinuria were considered to be steroid resistant.

### Measurement of blood lipids, albumin and urine protein

Fasting levels of venous blood from each experimental group were collected, and blood lipids were analyzed. Serum albumin was measured using a bromocresol green assay, and total cholesterol, LDL cholesterol, triglyceride and 24-hour urine protein were measured on an AU5400 automatic biochemical analyzer using a colorimetric assay.

### Enzyme-linked immunosorbent assays (ELISAs)

Sandwich ELISA was used to detect serum CXCL16, IFN-γ and oxLDL levels according to the manufacturer’s instructions. Briefly, standard proteins and samples were diluted with standard diluent buffer, and 100 μl of standards, controls and diluted samples was added to the appropriate microtiter wells. The wells were covered and incubated for two hours at room temperature. Biotin-conjugated detection antibody (100 μl) (Life Technologies, CA, USA) was added into each well after washing with PBS, and incubation was continued for one hour at room temperature followed by another PBS wash step. Anti-rabbit IgG-HRP (100 μl) was then added to each well, and then the wells were covered and allowed to incubate for another 30 minutes at room temperature. Following another wash step, stabilized chromogen (100 μl) was added to each well, and incubation was continued for 30 minutes at room temperature in the dark. Lastly, stop solution was added to each well, and the absorbance of each well at 450 nm was recorded by a microtiter plate reader. A smooth standard curve was drawn according to standard protein OD values. Unknown sample protein concentrations were derived from this standard curve.

### Isolation of mononuclear cells from peripheral blood

Peripheral blood was diluted with PBS and then gently layered on top of Ficoll-Paque Plus solution (GE Healthcare Biosciences, PA, USA) followed by centrifugation for 30 minutes at 1,600 rpm at room temperature. Peripheral blood mononuclear cells (PBMCs) were collected from the interface between the Ficoll-Paque layer and the diluted plasma layer and transferred into a new tube, centrifuged and washed twice with PBS. The cells were then resuspended in PBS and separated into aliquots for further analysis.

### Detection of the T cell subpopulation and NK cells with flow cytometry

Viable mononuclear cells (1 × 10^6^) were suspended in 400 μl of cold FACS solution per sample. Prediluted antibody mixture (100 μl) (BD company, NJ, USA) was added to all samples and mixed by pipetting. After incubating on ice for 45 minutes, the stained cells were centrifuged and resuspended in 150 μl of FACS fix solution prior to flow cytometric detection.

### Statistical analysis

GraphPad Prism software (version 5) was used for data analysis. Measurement data was described as mean ± standard deviation. One-way analysis of variance was used to compare means among three groups, while the Newman-Keuls test was used to compare means between any two groups. The Spearman’s two-tail test was used for correlation analysis. Significance was set at P < 0.05.

## Results

### Serum CXCL16 increased in active nephrotic syndrome

**Figure 1:**
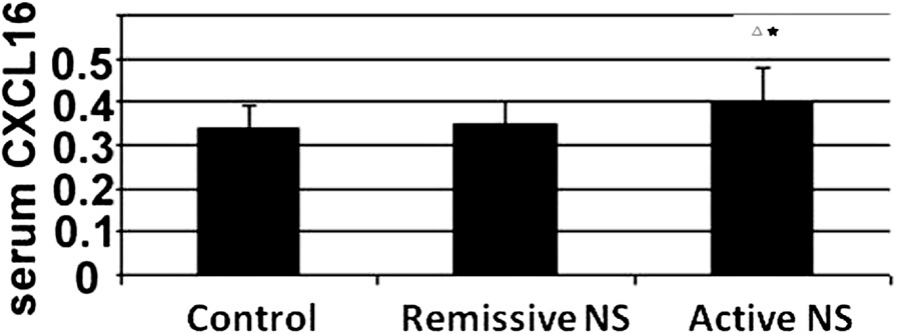
**Serum CXCL16 increased in active nephrotic syndrome.** p < 0.05(^△^) vs Control; p < 0.05(^★^) vs Remissive NS.

We detected an increase in the levels of serum CXCL16 in patients with active NS relative to those with remissive NS and the control group. As shown in Figure 1, serum CXCL16 in patients with active NS was calculated to be 0.40 ± 0.08 ng/ml, significantly higher than that of remissive NS patients (0.35 ± 0.05 ng/ml) and the control group (0.34 ± 0.05 ng/ml) ( P < 0.05). These results suggested that serum CXCL16 is increased in patients with active NS.

### Serum CXCL16 is positively correlated with the concentration of blood lipids in active NS patients

Hyperlipidemia is an important pathophysiological characteristic of children with primary NS. We therefore assessed the correlation between serum CXCL16 and the concentration of blood lipids. The results shown in Table 1 suggested that the concentration of blood total cholesterol (CHOL), triglycerides (TG), low-density lipoprotein cholesterol (LDL-C) and oxLDL in the active NS group is significantly higher than that in the remissive NS and control groups (P < 0.01). There was no statistically significant difference in the concentration of blood lipids between the remissive NS and control groups, consistent with previous research. Correlation analysis revealed that serum CXCL16 is positively correlated with CHOL, TG, LDL-C and oxLDL, having correlation coefficients (r) of 0.796, 0.646, 0.725 and 0.740, respectively, in the active NS group (P < 0.01). These results suggested that serum CXCL16 is positively correlated with the concentration of blood lipids in active NS patients.

**Table 1:** Blood lipid, albumin and 24-hour urine protein measurement (mean ± SD)

	**Active NS**	**Remissive NS**	**Control**
CXCL16	0.40 ± 0.08^★△^	0.35 ± 0.06	0.34 ± 0.05
CHOL (mmol/L)	10.65 ± 3.99^★★△△^	4.74 ± 1.31	3.90 ± 0.82
TG (mmol/L)	3.06 ± 1.15^★★△△^	1.23 ± 0.52	1.01 ± 0.40
LDL-C (mmol/L)	7.61 ± 2.91^★★△△^	3.01 ± 0.85	2.53 ± 0.66
oxLDL (μg/dL)	16.80 ± 2.30^★★△△^	13.88 ± 2.95	12.15 ± 1.44
Albumin (μg/dL)	17.31 ± 5.21^★★△△^	43.00 ± 2.49	44.08 ± 1.06
24-hour urine protein (g/24 h)	3.27 ± 1.12	——	——

p < 0.05(^△^), p < 0.01(^△△^) vs Control; p < 0.05(^★^), p < 0.01(^★★^) vs Remissive NS.

### Serum CXCL16 is positively correlated with 24-hour urine protein but negatively correlated with albumin in active NS patients

Leakage of protein from the blood into the urine is the major characterizing disorder of NS. We therefore assessed the correlation between serum CXCL16 and 24-hour urine protein and albumin. The results shown in Table 1 demonstrated that 24-hour urine protein in the active NS group was positive, with a value of 3.27 ± 1.12 g, and 24-hour urine protein in the remissive NS and control groups was negative. Albumin in the active NS group was significantly lower than that in the remissive NS and control groups (P < 0.01). There was no significant difference in 24-hour urine protein or albumin between the remissive NS and control groups (P > 0.05), consistent with previous research. Correlation analysis showed that 24-hour urine protein was positively correlated with CHOL, TG, LDL-C and oxLDL in active NS patients, having correlation coefficients of 0.514, 0.631, 0.516 and 0.641, respectively (P < 0.05). Serum CXCL16 was positively correlated with 24-hour urine protein but negatively correlated with albumin in active NS patients, having correlation coefficients of 0.736 and −0.628, respectively (P < 0.01).

### Serum CXCL16 is positively correlated with IFN-γ in active NS patients

**Figure 2:**
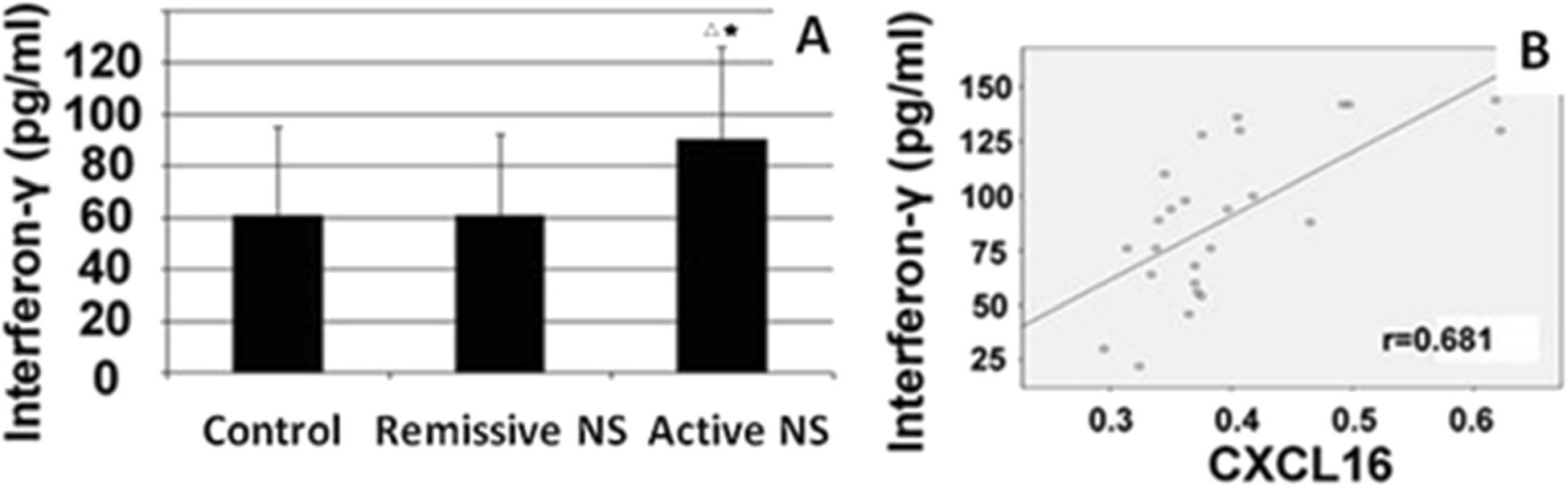
**Serum CXCL16 is positively correlated with IFN-γ in active NS patients. (A)** Serum IFN-γ level in different groups. **(B)** Correlation analysis between serum CXCL16 and IFN-γ in active NS patients. p < 0.05(^△^) vs Control; p < 0.05(^★^) vs Remissive NS.

Inflammation characterized by elevated inflammatory cytokines is common in NS. We therefore detected the pro-inflammatory protein IFN-γ in serum. As shown in Figure 2A, IFN-γ was significantly higher in the active NS group (90.12 ± 35.91 pg/ml) than in the remissive NS group (61.00 ± 30.97 pg/ml) and control group (61.00 ± 34.19 pg/ml) (P < 0.05). Correlation analysis revealed serum IFN-γ to be highly correlated with CHOL, TG, LDL-C, oxLDL, 24-hour urine protein and albumin in active NS patients, having correlation coefficients of 0.806, 0.767, 0.783, 0.825, 0.739 and −0.683, respectively (P < 0.01). Serum IFN-γ was also positively correlated with CXCL16 in active NS patients, having a correlation coefficient of 0.681 (P < 0.01, Figure 2B).

### Serum CXCL16 is positively correlated with CXCR6+ T cells in active NS patients

**Figure 3:**
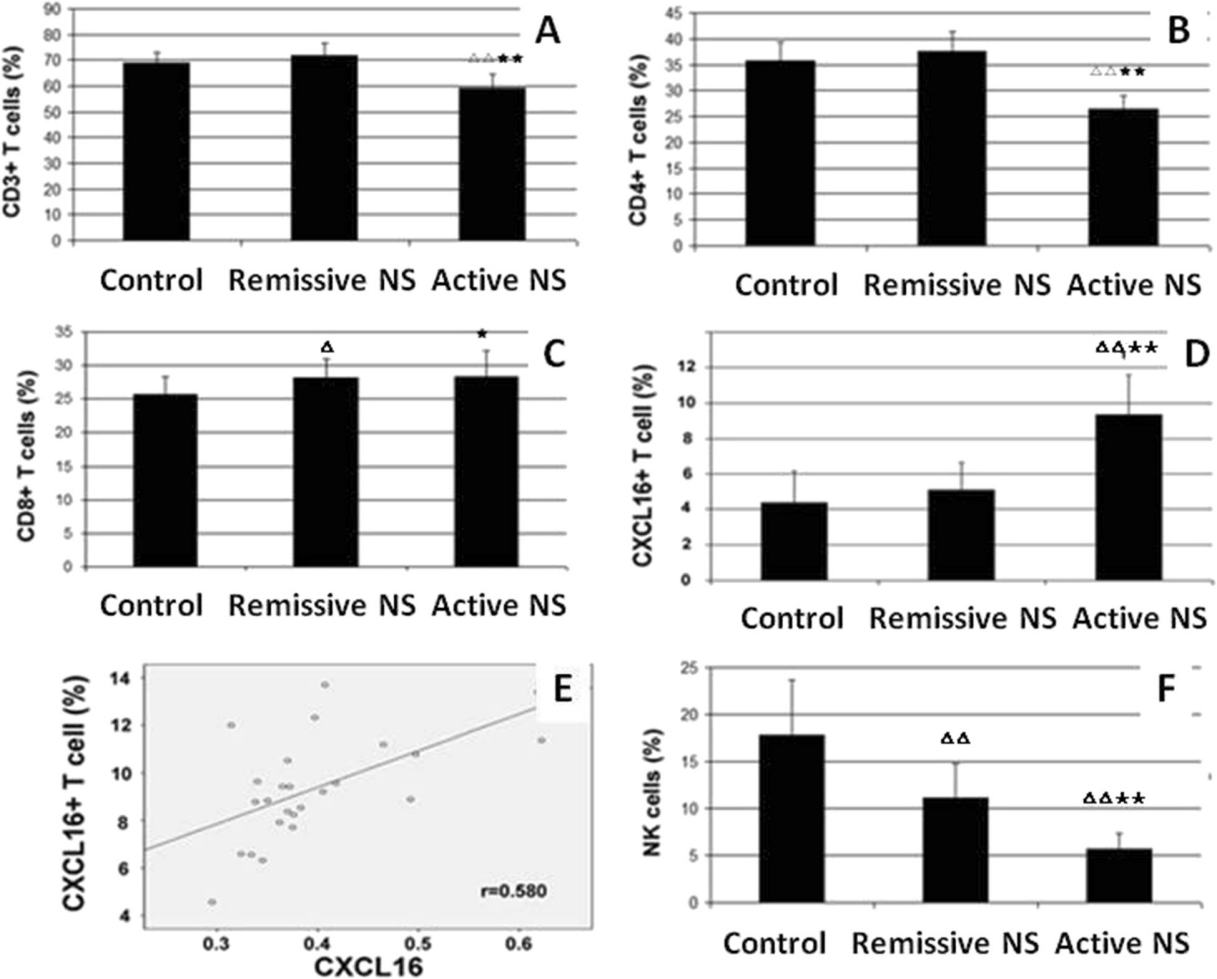
**Serum CXCL16 is positively correlated with CXCR6+ T cells in active NS patients. (A)** The percentage of CD3+ T cells in different groups. **(B)** The percentage of CD4+ T cells in different groups. **(C)** The percentage of CD8+ T cells in different groups. **(D)** The percentage of CXCR6+ T cells in different groups. **(E)** Correlation analysis between serum CXCL16 and CXCR6+ T cells in active NS patients. **(F)** The percentage of NK cells in different groups. p < 0.05(^△^), p < 0.01(^△△^) vs Control; p < 0.05(^★^), p < 0.01(^★★^) vs Remissive NS.

IFN-γ is a proinflammatory cytokine produced by T cells and natural killer (NK) cells. We therefore detected alterations in the T cell subpopulation and NK cells in active NS patients. The results showed that the percentage of CD3+ T cells and CD4+ T cells in the active NS group (59.31 ± 5.02% and 26.52 ± 2.62%, respectively) was significantly decreased compared with the control group (68.85 ± 4.09% and 35.63 ± 3.74%, respectively) (P < 0.01). The percentage of CD3+ T cells and CD4+ T cells in the remissive NS group was 71.90 ± 4.75% and 37.57 ± 3.76%, respectively, but there was no statistically significant difference when compared with the control group (P > 0.05, Figure 3A and 3B). The percentage of CD8+ T cells in the active NS group (28.35 ± 3.90%) and the remissive NS group (28.17 ± 2.77%) was higher than that in the control group (25.67 ± 2.66%) (P < 0.05, Figure 3C). We also detected the percentage of CXCL16+ T cells in each group, and the results showed that CXCL16+ T cells were significantly increased in the active NS group (9.36 ± 2.22%) compared with the remissive NS group (5.10 ± 1.53%) and control group (4.39 ± 1.76%) (P < 0.01, Figure 3D). Correlation analysis showed that serum CXCL16 was highly correlated with the number of CXCR6+ T cells (r = 0.681, P < 0.01, Figure 3E). To our surprise, the percentage of NK cells in the active NS group (5.68 ± 1.69%) and the remissive NS group (11.15 ± 3.66%) was significantly decreased compared with the control group (17.74 ± 5.91%) (P < 0.01, Figure 3F).

## Discussion

Hyperlipidemia is an important pathophysiological change in children with primary NS. In 1982, Moorhead et al. proposed the theory of lipid nephrotoxicity resulting from abnormalities of lipid metabolism in chronic kidney disease [3]. The results of many clinical and animal models strongly support this hypothesis [13-16]. Lee et al. found foam cells, lipid deposits and oxLDL in renal biopsies of focal segmental glomerulosclerosis (FSGS) [17]. Proteinuria and FSGS in rats with puromycin nephropathy were shown to worsen with cholesterol intake [18], and Joles et al. found that podocytes were an early target of kidney injury in animals on a high-fat diet [19]. Therefore, hyperlipidemia and lipid deposition in the kidney are very important risk factors for glomerulosclerosis.

This study indicates that the levels of blood total cholesterol, triglycerides, LDL-C and oxLDL in the active NS group were significantly higher than those in the remissive NS and normal control groups, implying metabolic disorder of blood lipids during NS activity. Lipids can stimulate interactions between mesangial cells and monocytes and induce monocytes to secrete large amounts of inflammatory cytokines, such as IL-6, PDGF-1 and TGFβ1 [3]. The IL-1β has been shown to induce the expression of scavenger receptors, increase cholesterol levels in vascular smooth muscle cells, mesangial cells and macrophages, interfere with feedback regulation of LDL receptors leading to continuous uptake of LDL, and promote the formation of foam cells [20,21]. oxLDL is a major contributor to foam cell formation and may also cause injury to endothelial cells and promote the proliferation of vascular smooth muscle cells during the development of atherosclerosis. oxLDL is a powerful chemokine of macrophages and T lymphocytes that can recruit circulating monocytes through direct or indirect induction of chemokine and adhesion molecule production in smooth muscle cells, mesangial cells and/or endothelial cells [22,23]. In addition, in vitro assays identified certain cytotoxic side effects of oxLDL, including apoptosis of podocytes [23]. In this study, correlation analysis showed that levels of blood total cholesterol, triglycerides, LDL-C and oxLDL were positively correlated with 24-hour urine protein in the active NS group, suggesting that dyslipidemia may be involved in the occurrence of NS. However, reports of the participation of oxLDL in the occurrence of primary NS in children are rare.

In this study, we showed a significant increase in the levels of serum IFN-γ in children with active simple-type NS relative to the remissive NS and normal control groups. Furthermore, serum IFN-γ in the active NS group was positively correlated with 24-hour urine protein and negatively correlated with plasma albumin. These results indicate that IFN-γ may be involved in the pathogenesis of idiopathic NS and associated with NS activity. Notably, serum IFN-γ in children with active NS was also positively correlated with levels of blood total cholesterol, triglycerides, LDL-C and oxLDL, indicating that IFN-γ may be involved in NS dyslipidemia and promote lesion inflammation.

Reports of CXCL16 in the development of inflammation in kidney disease are few. While screening for potential biomarkers of lupus nephritis, Tianfu Wu et al. [16] discovered CXCL16 protein in the urine of mice with spontaneous lupus nephritis. Notably, the presence of CXCL16 correlated with the period of disease activity. In addition, increased CXCL16 was found in the urine of patients with lupus nephritis and was significantly associated with urinary protein levels as well as activity index and score of systemic lupus erythematosus. Xia Y et al. [24] found that CXCL16 knockout mice were protected from angiotensin II-induced renal dysfunction, proteinuria, and fibrosis, and proved that CXCL16 plays a pivotal role in the pathogenesis of hypertensive kidney injury and fibrosis through regulation of macrophage and T cell infiltration and bone marrow-derived fibroblast accumulation. However, few studies have focused on the association of CXCL16 alteration in children with primary NS.

Schramme et al. not only found that CXCL16 was expressed in human mesangial cells (hMCs), but also confirmed that a mixture of cytokines (IFN-γ, TNF-α and IL-1β) could further increase the expression of CXCL16 [25]. Through the stimulation of cultured human podocytes in vitro using IFN-γ, TNF-α and angiotensin II, Gutwein et al. found that IFN-γ and TNF-α could increase the expression of podocyte transmembrane and soluble CXCL16, while angiotensin II stimulation had no effect on CXCL16 expression [26]. Wagsater et al. investigated the effect of IFN-γ, TNF-α, IL-12 and other cytokines on the expression of CXCL16 [10]. Their results indicated that IFN-γ was the strongest stimulating factor for CXCL16 expression, up-regulating levels of CXCL16 mRNA as well as transmembrane and soluble forms of the protein. Our study showed that both serum CXCL16 and IFN-γ in the active NS group were significantly increased. Furthermore, correlation analysis revealed a positive correlation between levels of serum CXCL16 and IFN-γ, suggesting that increased IFN-γ in active simple-type NS may be a stimulus for elevated serum CXCL16. However, the exact mechanism of this activity requires further study.

Shalhoub showed that circulating lymphatic factors could result in damage to the glomerular basement membrane and thereby proposed that NS might be associated with T-cell immune abnormalities [27]. Schramme et al. found that acute tubular injury was associated with increased CXCL16 in renal transplant urine and related to increased CXCL16 in the distal tubule and collecting duct [28]. Currently, CXCR6 is the only known receptor of CXCL16. CXCR6 is primarily expressed in naive CD8+ T cells, activated CD4+ T cells, activated CD8+ T cells, NK cells and polymorphonuclear neutrophils [6,29]. Chemotaxis assays indicated that only cells with high expression of CXCR6 could respond to CXCL16 [30]. Using a rat nephritis model with anti-glomerular basement membrane antibody, Garcia et al. found that CXCL16 was expressed in glomerular endothelial cells and regulated macrophage adhesion [13]. During acute inflammation, blockage of CXCL16 significantly inhibited monocyte/macrophage infiltration and reduced glomerular damage and proteinuria; therefore, CXCL16 and CXCR6 may play an important role in the migration of T cells [5]. It is suggested that T cell migration into inflammatory lesions is mediated by the specific interaction of CXCL16 and CXCR6. Previous studies confirmed that CXCR6 and secreted soluble CXCL16 might be responsible for inducing chemotactic migration of proinflammatory cells into arthritic joints and sites of liver inflammation [31-34]. However, there are few reports regarding CXCR6 induction of chemotactic migration of inflammatory cells in renal lesions of childhood NS.

Our results showed that the ratio of peripheral blood CD3+, CD4+, CD4+ 8+ and NK (CD16+ 56+) T cells was significantly lower in the active NS group than in the remissive NS and normal control groups. Furthermore, active NS group CD8+ T cells were significantly higher in number than in the control group, suggesting that the ratio between T lymphocyte subsets was unbalanced during primary NS activity, consistent with the reported literature. However, CD8+ T cells were still more numerous in the remissive group than in the normal control group, and NK (CD 16+ 56+) T cells in the remissive group were fewer in number than in the control group. Peripheral blood CXCR6+ T cell numbers in the active NS group were significantly higher than in the remissive NS and normal control groups, while peripheral blood CXCR6+ T cell numbers in the remissive NS group were no different than the normal control group. Moreover, correlation analysis revealed that the levels of serum CXCL16 in the active NS group were positively correlation with the quantity of peripheral blood CXCR6+ T cells, suggesting that CXCR6 and soluble CXCL16 may participate together in the occurrence of NS. Further study is needed to confirm the level of involvement of CXCL16 and CXCR6 in the migration of inflammatory cells to renal lesions.

In summary, we found that serum CXCL16 is increased in patients with active NS and is correlated with blood lipids, 24-hour urine protein and immune and inflammation responses, suggesting that CXCL16 may serve as a useful index or biomarker for disease activity in children with nephrotic syndrome.

## Competing interests

The authors declare that they have no competing interests.

## Authors’ contributions

Study concept and design: SS. Acquisition of data: QL, JZ. Analysis and interpretation of data: QL, JZ. Drafting of the manuscript: JZ, QL, SS. Critical revision of the manuscript for important intellectual content: SS. Statistical analysis: YZ, JZ. Administrative, technical, or material support: JZ, QL, YZ, XY, LW, AZ. Study supervision: SS. All authors read and approved the final manuscript.
